# Effects of Methyl Donor Diets on Incisional Pain in Mice

**DOI:** 10.1371/journal.pone.0077881

**Published:** 2013-10-24

**Authors:** Yuan Sun, Deyong Liang, Peyman Sahbaie, J. David Clark

**Affiliations:** 1 Department of Anesthesiology, Stanford University School of Medicine, Stanford, California, United States of America; 2 Department of Anesthesiology, Veterans Affairs Palo Alto Health Care System, Palo Alto, California, United States of America; University of Arizona, United States of America

## Abstract

**Background:**

Dietary supplementation with methyl donors can influence the programming of epigenetic patterns resulting in persistent alterations in disease susceptibility and behavior. However, the dietary effects of methyl donors on pain have not been explored. In this study, we evaluated the effects of dietary methyl donor content on pain responses in mice.

**Methods:**

Male and female C57BL/6J mice were treated with high or low methyl donor diets either in the perinatal period or after weaning. Mechanical and thermal nociceptive sensitivity were measured before and after incision.

**Results:**

Mice fed high or low methyl donor diets displayed equal weight gain over the course of the experiments. When exposed to these dietary manipulations in the perinatal period, only male offspring of dams fed a high methyl donor diet displayed increased mechanical allodynia. Hindpaw incision in these animals caused enhanced nociceptive sensitization, but dietary history did not affect the duration of sensitization. For mice exposed to high or low methyl donor diets after weaning, no significant differences were observed in mechanical or thermal nociceptive sensitivity either at baseline or in response to hindpaw incision.

**Conclusions:**

Perinatal dietary factors such as methyl donor content may impact pain experiences in later life. These effects, however, may be specific to sex and pain modality.

## Introduction

Epigenetic mechanisms (DNA methylation, histone modification and non-coding RNA regulation) include processes that lead to stable and/or heritable changes in gene function without concomitant changes in DNA sequence [1]. Both human and animal studies have demonstrated that dietary, lifestyle and other environmental factors influence epigenetic marks and processes which have important consequences on disease susceptibility and behavior [2].

Perinatal dietary supplementation with methyl donors such as folate, methionine, betaine and choline has been found to be effective in promoting DNA methylation resulting in changes in various phenotypes in the offspring [3]. High methyl donor diet in the perinatal period shifts offspring coat color from yellow to brown due to CpG hypermethylation at the agouti viable yellow (A^vy^) locus [4]. Similarly 82 gene-associated loci were differently methylated in the offspring of dams fed high versus low methyl donor diets [5]. In these experiments changes in DNA methylation caused aberrant gene transcription and enhanced the severity of allergic airway disease in the offspring [5]. Susceptibility to colitis was increased as well [6]. The methylation of DNA in older individuals can also be modified by nutritional interventions. For example, the hypomethylation of genomic DNA in lymphocytes in postmenopausal women is reversed by increasing dietary folate [7]. Similarly, increased folic acid intake during the juvenile period increases DNA methylation and reverses the metabolic dysregulation caused by perinatal dietary protein restriction [8].

Epigenetically regulation of gene expression in nociceptive pathways is known to contribute to the induction and maintenance of pain sensitization. Wang et al. found that administration of a DNA methyltransferase inhibitor blocked global DNA methylation and alleviated neuropathic pain in rats [9]. Moreover, the methylation patterns of many pain related genes are controlled by DNA methylation, such as SPARC (secreted protein, acidic, rich in cysteine) [10], [11], MOR (µ-opioid receptor) [12], and EDNRB (encoding the endothelin B receptor) [13]. In the present study, we aimed to investigate whether dietary methyl donor content would affect the nociceptive responses when given in the perinatal period or after weaning.

## Methods

### Animal Use

All experimental protocols were reviewed and approved by Veterans Affairs Palo Alto Healthcare System Institutional Animal Care and Use Committee prior to beginning the work. Mice were housed four per cage and maintained on a 12-h light/dark cycle and an ambient temperature of 22±1°C, with food and tap water available *ad libitum*.

### Methyl Donor Diet and Breeding Protocol

High and low methyl donor chows were nutritionally complete and were purchased from Research Diets (New Brunswick, NJ). The high methyl donor diet was supplemented with folic acid, vitamin B12, choline, L-methionine, zinc, betaine and genistein as used in our previous experiments [14]. The differences in the contents of the methylation-related components of all the diets were shown in Table S1.

For the perinatal methyl donor supplementation studies, C57BL/6J male and female mice were purchased from the Jackson Laboratory (Bar Harbor, ME) at 5 weeks age. Females were randomly divided into two groups to receive high or low methyl donor diets. The diets were provided for 2 weeks prior to mating and throughout gestation and lactation [4], [5], [15]. At P21, the pups were weaned and received the standard rodent diet (2018, Harlan-Teklad, Madison, WI). This diet was continued during their use in experiments which began at 8 weeks of age.

For the postweaning methyl donor supplementation studies, C57BL/6J dams with P14 pups were purchased from the Jackson Laboratories. These mice received standard rodent diet after arrival at our institution. At P21, the pups were weaned and randomized to receive either high or low methyl donor diets. The diets were provided until they were used in behavioral tests at 8 weeks of age, and continued through the testing period. Thus at the time of completion of testing, each animal had been exposed to either the high or low methyl donor diet for approximately 6 weeks either in the perinatal period or later in life.

### Hindpaw Incision

The hindpaw incision model in mice was performed in our laboratory as described in previous studies [16]. Briefly, under isoflurane anesthesia, a 5 mm longitudinal incision was made with a #11 scalpel on the plantar surface of the right hindpaw. The incision was sufficiently deep to divide deep tissue including the plantaris muscle longitudinally. After controlling bleeding, a single 6-0 nylon suture was placed through the midpoint of the wound.

### Nociceptive Testing

All nociceptive testing was done with the experimenter blind to diets treatment.

### Mechanical Allodynia

Mechanical nociceptive thresholds were assayed using von Frey filaments according to a modification of the “up-down” algorithm as described in previous studies [16]. Fibers of sequentially increasing stiffness with initial bending force of 0.2 gram were applied to the plantar surface of the hindpaw adjacent to the incision, just distal to the first set of foot pads and left in place 5 sec with enough force to slightly bend the fiber. Withdrawal of the hindpaw from the fiber was scored as a response. When no response was obtained, the next stiffer fiber in the series was applied in the same manner. If a response was observed, the next less stiff fiber was applied. Testing proceeded in this manner until 4 fibers had been applied after the first one causing a withdrawal response allowing the estimation of the mechanical withdrawal threshold using a curve fitting algorithm [17].

### Thermal Hyperalgesia

Paw withdrawal response latencies to noxious thermal stimulation were measured using the method of Hargreaves et al [18]. Mice were placed on a temperature-controlled glass platform (29°C) in a clear plastic enclosure. After 30 min of acclimation, a beam of focused light was directed towards the same area of the hindpaw as described for the von Frey assay. In these experiments, the light beam intensity was adjusted to provide an approximate 10 s baseline latency in control mice.

### Statistical Analysis

All data are expressed as mean ± SEM. Analysis of repeated parametric measures was accomplished using a two-way analysis of variance followed by Bonferroni testing. For simple comparisons of two groups, a two-tailed Student’s *t* test was employed. *P* values less than 0.05 were considered significant (Prism 5; GraphPad Software, La Jolla, CA).

## Results

### Mice Fed High or Low Methyl Donor Diets displayed Equal Weight Gain over the Course of the Experiments

We first determined if significant differences in growth rates were present in mice treated with high and low donor diets. There was no significant difference in body weight between dietary groups in male and female mice under both perinatal and postweaning treatment protocols (Table 1). Additionally, we did not observe developmental abnormalities or behavior suggestive of motor discoordination.

**Table 1:** Body weight for mice fed high versus low methyl donor diet.

Sex	Males		Females	
	low	high	low	high
Perinatal	26.53±0.58	25.40±0.49	20.17±0.36	19.42±0.46
Postweaning	23.24±0.63	22.25±0.68	18.16±0.28	18.51±0.32

The weights of the male and female C57BL/6 mice were determined at 8–9 weeks of age when behavior testing began. n = 8/group. Data are displayed in grams.

### Perinatal Supplementation of Dietary Methyl Donors Increased Mechanical Nociceptive Sensitization in Males

**Figure 1:**
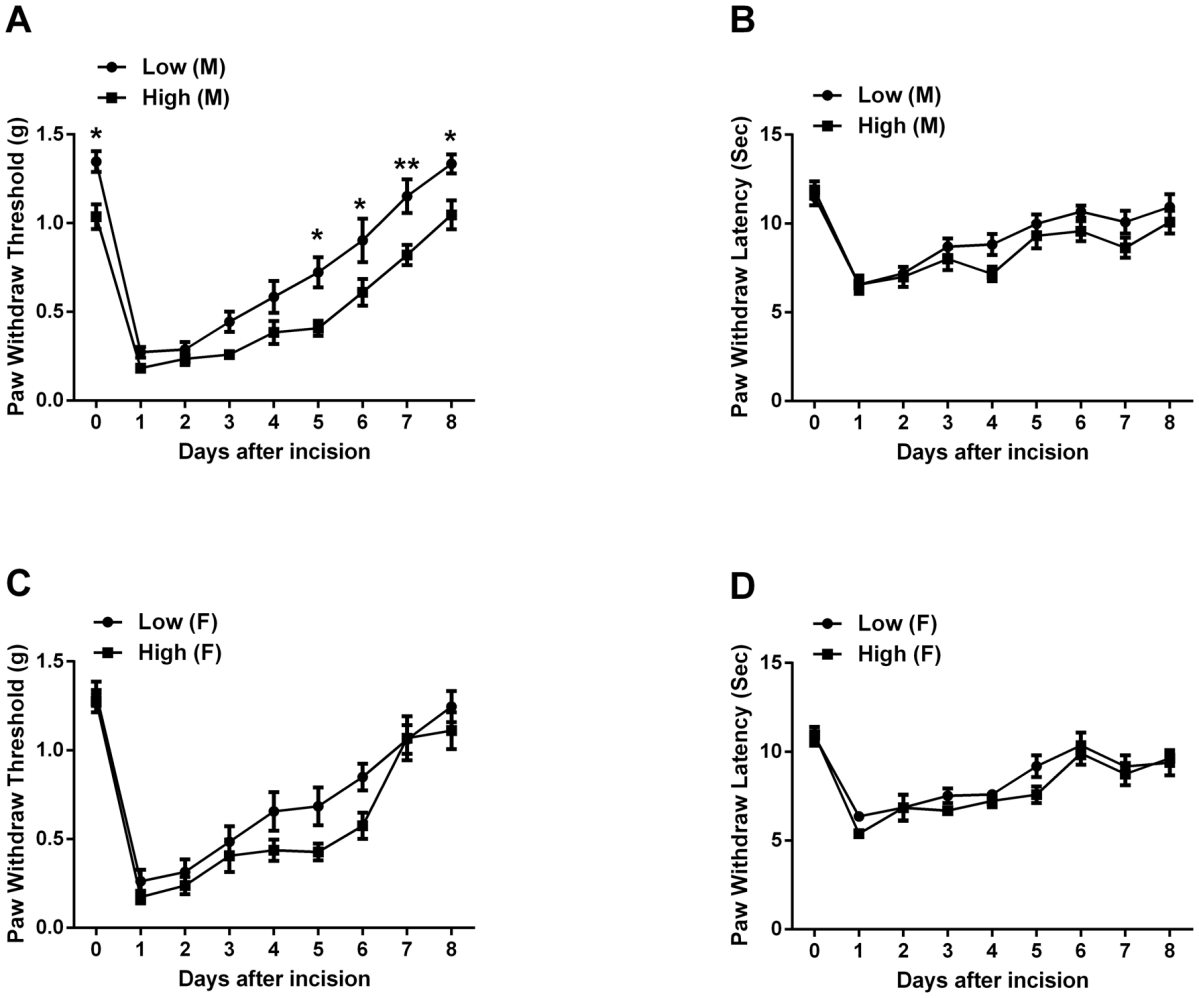
Mechanical and thermal nociceptive sensitivity for mice fed high versus low methyl donor diets in the perinatal period. In panels A and B the mechanical and thermal sensitivity are displayed for male offspring. Panels C and D the mechanical and thermal sensitivity are displayed for female offspring. Values are displayed as the mean ± SEM. n = 14/group. M, male; F, female. *p<0.05 or **p<0.01 versus low methyl diet treatment.

In male offspring, there was a significant reduction in baseline mechanical nociceptive threshold in the perinatal high methyl donor group (Fig. 1A). This enhanced sensitivity was observed throughout the post-incisional period (Fig. 1A). No differences in thermal sensitivity at were observed either baseline or after incision (Fig. 1B). In contrast to male offspring, no significant differences between dietary groups were noted in nociceptive sensitivity to thermal and mechanical stimuli before or after incision in female offspring (Fig. 1C and 1D), though the high methyl diet trended towards enhanced sensitivity after incision.

**Figure 2:**
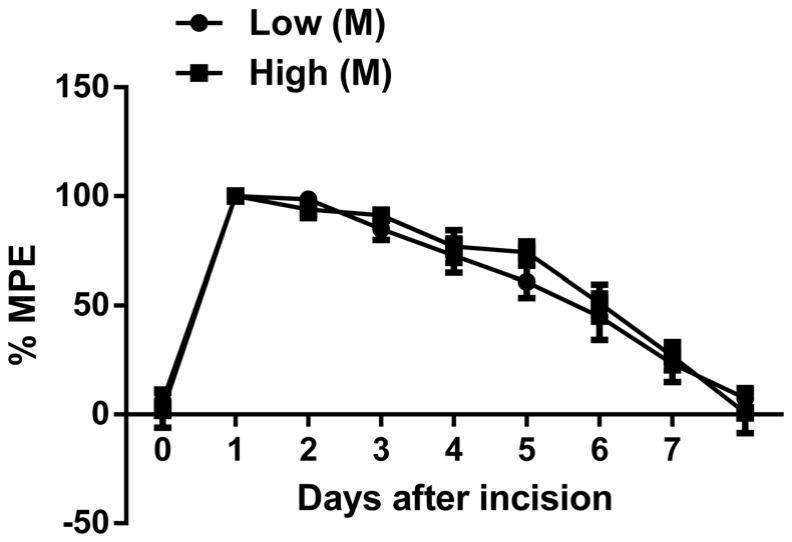
Normalized mechanical nociceptive sensitization curves for male mice fed high versus low methyl donor diets in the perinatal period. Data are presented as % maximum possible effect (% MPE) for each set of dietary data. For each diet, % MPE = 100*(baseline-measured)/(baseline-minimum). Values are displayed as the mean ± SEM, n = 14/group. M, male.

We next determined if the effects observed in male mice after incision could be attributed to baseline differences alone. When mechanical nociceptive threshold responses were normalized to their respective baseline values, no significant differences between the two dietary groups were observed between the male mice (Fig. 2). The value of areas under the curve nociceptive threshold vs. time curves was 492.1 versus 515.8 units in low and high methyl diet group, respectively.

### Postweaning Supplementation of Dietary Methyl Donors does not alter Nociceptive Sensitization

**Figure 3:**
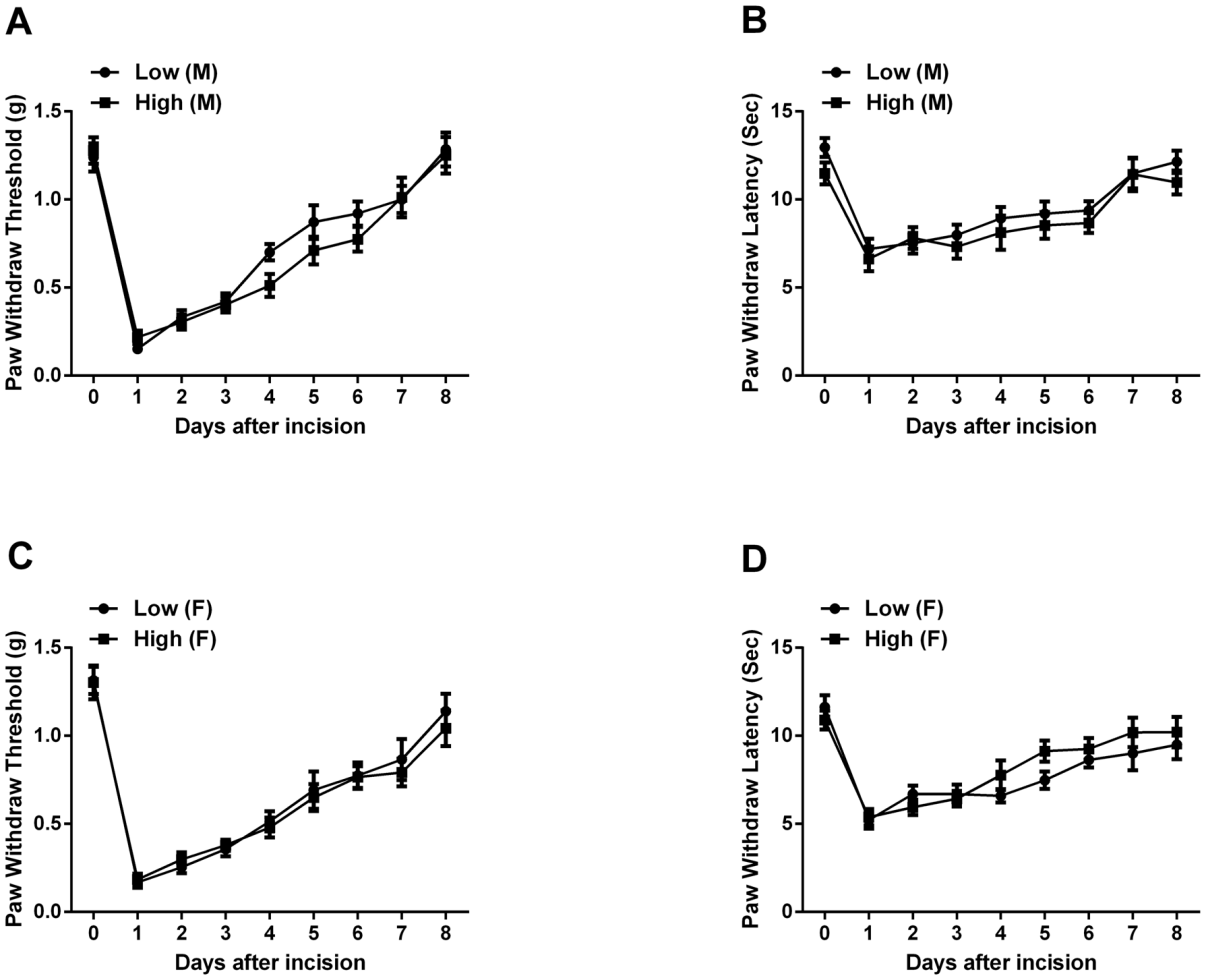
Mechanical and thermal nociceptive sensitivity for mice fed high versus low methyl donor diets after weaning. In panels A and B the mechanical and thermal sensitivity are displayed for male. Panels C and D the mechanical and thermal sensitivity are displayed for female. Values are displayed as the mean ± SEM. n = 8/group. M, male; F, female.

The same behavior testing protocol described for the perinatally treated mice was used on the mice starting methyl donor dietary treatments after weaning. We observed no significant differences in mechanical and thermal nociceptive sensitization at baseline and in response to incision between dietary groups for both genders (Fig. 3).

## Discussion

Recent studies suggest early life dietary factors through epigenetic mechanisms modulate adult diseases [19], [20]. Maternal diet methyl donor content plays a vital role in determining DNA methylation in the offspring. In addition, emerging evidence has confirmed that epigenetic factors including DNA methylation control nociceptive sensitivity [9], [21]. In our experiments, we found that methyl donor depletion or supplementation in the diet did not lead to gross differences in growth or development in mice whether the diet was applied at early or later life. However, perinatal methyl donor content did alter nociceptive thresholds in a sex-dependent manner.

Several pain and analgesia-related genes are thought to be controlled by DNA methylation. For example, chronic opioid administration is associated with increased DNA methylation at the LINE-1 global methylation site as well as increased methylation of a CpG island in the µ-opioid receptor promoter. The level of LINE-1 methylation was correlated in these studies to pain levels [12]. Additional studies have demonstrated that expression of pain-related genes such as SPARC (secreted protein, acidic, rich in cysteine) [11], EDNRB (encoding the endothelin B receptor) [13], DPP4 (dipeptidyl peptidase-4) [22] and others are also under the control of methylation-related epigenetic mechanisms. Moreover, global DNA methylation was increased in spinal cord tissue after sciatic nerve chronic constriction injury, and mechanical allodynia was markedly attenuated by a DNA methyltransferase inhibitor [9]. Large numbers of genes in various tissues (skin, sensory neurons, spinal cord and brain) have been demonstrated to contribute to determining nociceptive sensitivity [23]. Future studies broadly examining which of these genes are controlled by dietary methyl donor content might provide a mechanistic explanation for the differences we observed.

Sex-dependent effects of dietary methyl donor content have been observed by other investigators, and male offspring appear to be more often affected. A diet deficient in methyl donors during the perinatal period alters glucose homeostasis in rats, but only in the adult male offspring [24]. More broadly, Sinclair et al. studied the methylation status of 1400 CpG islands in the offspring of sheep fed a methylation-promoting periconceptional diet [25]. The authors found that more than half of the loci affected by diet in the adult offspring were specific to males. Importantly, the diet fed to the sheep affected the weight, immune status, insulin resistance and blood pressure of the male offspring to a greater degree than female offspring.

Methyl donors, such as folic acid, choline, vitamin B12 can be found in various prenatal vitamins and supplements consumed by humans, however, intake of excessive methyl donors might lead to some unexpected biological and pathological consequences. For example, supplementation of maternal diet with methyl donors was associated with greater levels of inflammation in airway and intestine in mice offspring [5], [6]. Using similar dietary treatments, we found that high methyl donor content caused more mechanical sensitivity in male offspring. It is unclear at this time whether use of these supplements has any effect on pain sensitivity in human offspring. Therefore, identification of the epigenetic role of methyl donor and the functional significance of the regulated genes in specific tissue will be important goals.

Epigenetic mechanisms are felt to regulate development, physiology and behavior. Aggressive efforts are underway to develop drugs targeting epigenetic mechanisms for the control of cancer and other diseases. Our results and those of others suggest that dietary factors may control a range of phenotypes perhaps through the control of DNA methylation occurring early in life. Since some methyl donors exhibit broad effects on susceptibility to diseases or treatment, it is possible that other mechanisms rather than epigenetic ones could contribute to phenotypic changes we observed. Fully understanding the types of pain affected by diet, the mechanisms of those effects and measures that can be taken to augment or reduce diet-induced pain-related changes are priorities for our future investigation.

## Supporting Information

Table S1 Differences in contents of the methylation-related components of the diets. Low and high methylation diets were formulated by Research Diets (New Brunswick, NJ); Jackson Laboratories in-house diet was provided by LabDiet (St. Louis, MO); VA Palo Alto Health Care System in-house diet was provided by Teklad Diets (Madison, WI).
(DOCX)
