# Supplementary material for: Effects of Methyl Donor Diets on Incisional Pain in Mice
Source: PLoS One. 2013 Oct 24;8(10):e77881. doi: 10.1371/journal.pone.0077881 (PMC3812030; doi:10.1371/journal.pone.0077881)
Supplement: Table S1 — Differences in contents of the methylation-related components of the diets. Low and high methylation diets were formulated by Research Diets (New Brunswick, NJ); Jackson Laboratories in-house diet was provided by LabDiet (St. Louis, MO); VA Palo Alto Health Care System in-house diet was provided by Teklad Diets (Madison, WI). (DOCX) [file pone.0077881.s001.docx]

Table S1. Differences in contents of the methylation-related components of the diets

| Ingredient | | Low methylation diet (g/kg) | | High methylation diet (g/kg) | | Jackson Laboratories in-house diet (g/kg) | | VA Palo Alto Health Care System in-house diet (g/kg) | |
| --- | --- | --- | --- | --- | --- | --- | --- | --- | --- |
| Methionine | | 5.2 | | 12.7 | | 7.3 | | 4 | |
| Zinc Carbonate | | 0.03 | | 0.18 | | 0.085 | | 0.07 | |
| Folic acid | | 0.002 | | 0.017 | | 0.002 | | 0.004 | |
| Betaine | | 0 | | 15 | | 0 | | 0 | |
| Cyanocobalamin | | 25x10^-6^ | | 175x10^-6^ | | 50x10^-6^ | | 80x10^-6^ | |
| Choline | | 1 | | 15 | | 2 | | 1.2 | |
| Genistein | 0 | | 0.3 | | 0 | | 0 | |  |

Low and high methylation diets were formulated by Research Diets (New Brunswick, NJ); Jackson Laboratories in-house diet was provided by LabDiet (St. Louis, MO); VA Palo Alto Health Care System in-house diet was provided by Teklad Diets (Madison, WI).
